# Difference in post-stress recovery of the gut microbiome and its altered metabolism after chronic adolescent stress in rats

**DOI:** 10.1038/s41598-020-60862-1

**Published:** 2020-03-03

**Authors:** Mengyang Xu, Chen Wang, Kristen N. Krolick, Haifei Shi, Jiangjiang Zhu

**Affiliations:** 1 Department of Biology, Miami University, Oxford, OH 45056 USA; 2 Department of Human Sciences, The Ohio State University, Columbus, OH 43210 USA; 3 James Comprehensive Cancer Center, The Ohio State University, Columbus, OH 43210 USA

**Keywords:** Biotechnology, Microbiology, Medical research

## Abstract

The human gut microbiome plays a central role in human health, and has been implicated in the development of a number of chronic gastrointestinal and systemic diseases. For example, microorganisms can serve as microbial endocrine mediators and can respond to stimuli and produce neurochemicals, ultimately influencing the brain-gut-microbiome axis of their host, a bidirectional communication system between the central nervous system and the gastrointestinal tract, especially during developmental stages. To begin to explore potential dynamic changes of the gut microbiome, we characterized gut microbiota in adolescent rats that underwent a fixed period of restraint stress, examined whether the gut microbial population and their metabolic functions were changed by stress, and if such changes during adolescence persist or recover in young adulthood. Integrated 16S ribosomal DNA sequencing and liquid chromatography coupled tandem mass spectrometry (LC-MS/MS) based metabolic profiling were utilized to discover any significant differences in gut microbial genus and microbial metabolites immediately at the end of the chronic restraint stress and three weeks after the stress treatment, compared to control rats that did not receive stress treatment. Interestingly, while adolescent chronic stress-induced differences in relative microbial abundance (i.e., microbial species and distribution) disappeared three weeks after the stress treatment ended, the differences in microbial metabolic profiles persisted into adulthood. In addition, a number of significantly altered metabolites and their correlated gut microbes detected in our study facilitated a possible connection between gut microbiota and host stress response, which can be further investigated in the future to study the causal relationship between gut microbial metabolites and their impact on human health.

## Introduction

Stress is known as an important factor for both mental and physical health of humans^1–3^. Chronic stress causes dysregulation of the hypothalamic-pituitary-adrenal (HPA) axis, leading to an increased level of glucocorticoids that contribute to the development of neuropsychiatric disorders such as anxiety and depression, and metabolic disturbances such as obesity and insulin resistance^4,5^. Stress that occurs during adolescence, a crucial period of brain development and maturation, could potentially increases one’s susceptibility to development of neuropsychiatric disorders during adulthood and has immediate consequences on an individual’s psychological health^6^. Thus, it is important to study the effects of stress at the vulnerable time period of adolescence and its long term impact as one ages into adulthood.

Humans coexist in a mutualistic relationship with their gut microbiota. The human gut microbiota plays a vital role in human health, and has been implicated in the development of a number of chronic gastrointestinal and systemic diseases^7–9^. The human microbiome is composed of over 10 trillion microbial cells and can produce a diverse set of small molecules and bioactive compounds that can trigger host metabolic, nutritional, behavioral, and immune responses^10–12^. Human gut microbiota has been estimated to contain around 1, 000 bacterial species that express 100-fold more genes than the human genome^13^. Past studies have indicated that change in the gut microbiota population and the metabolic products of microbiota themselves can have both beneficial and detrimental effects on human and rodent health^7,14,15^. The gut microbiota can serve as microbial endocrine mediators and can respond to and produce circulating neurochemicals that influence physiological function and behavior of their host^16^. The brain-gut-microbiome axis is a bidirectional communication system between the central nervous system (CNS) and the microbiota of the gastrointestinal tract. Accumulating evidence reveals that gut microbiota and their metabolites can influenc the CNS through several channels^17^. Furthermore, a direct link between the HPA axis and gut microbiota has been reported in the literature^18^. For example, germ-free mice present elevated levels of corticosterone and adrenocorticotrophic hormone compared to conventionally raised mice^19^, suggesting increased activation of the HPA axis as a result of the absence of gut microbiota. In addition, the microbiota can even influence the CNS by directly mediating the neuronal activities of stress circuits^20^. Much can be learned about gut microbiota responses from the way they alter their metabolic function due to different environmental pressures^21^. Some microbial metabolites are substrates for neurochemicals that lead to long-term change in neurocircuits^22^.

In this study, we characterized gut microbiota and their metabolites in adolescent rats that underwent restraint stress through integrated 16S ribosomal DNA (rDNA) sequencing, liquid chromatography coupled tandem mass spectrometry (LC-MS/MS) based metabolic profiling, and correlative microbe–metabolite analysis to gain a better understanding of how gut microbiota respond under conditions of chronic host stress. We hypothesized that the gut microbial population and their metabolic functions would be changed by stress in adolescent rats, and such changes during adolescence might persist into adulthood. The next-generation sequencing technology, 16S rDNA amplicon sequencing has been widely used in the metagenomic analysis of gut microbiota^23^. LC-MS/MS has also been utilized as the state-of-the-art approach to measure the metabolic composition of biological samples for metabolic profiling studies^24,25^. Interrogation of both the microbial population and metabolites of the intestinal environment using these technical approaches provide valuable insight into gut microbe-host interactions.

## Materials and Method

### Animals

Male Sprague Dawley rats at the age of postnatal day (PD) 21 were purchased from Harlan Laboratories where dams are randomly allocated to have litters and the average litter size is 10–11 (Indianapolis, IN, USA). Rats were group shipped in craters with filtered coverings, and were single housed in autoclaved cages with autoclaved bedding and filtered cage lids that prevent exposure to pathogens at Miami University animal facility. All the rats of different treatment groups were randomly housed in the same room with a clean, stress-free environment under controlled lighting (12 h light: dark; lights on at 0600 and lights off at 1800) and temperature (21 °C). Pathogen status of the rats and the room was routinely checked. A standard rodent diet and water were provided *ad libitum* except during stress. The treatment groups stayed the same throughout the entire study. All animal work protocols were approved by the Institutional Animal Care and Use Committee (IACUC) at Miami University in accordance with the National Institutes of Health Guidelines for the Care and Use of Laboratory Animals.

**Figure 1:**
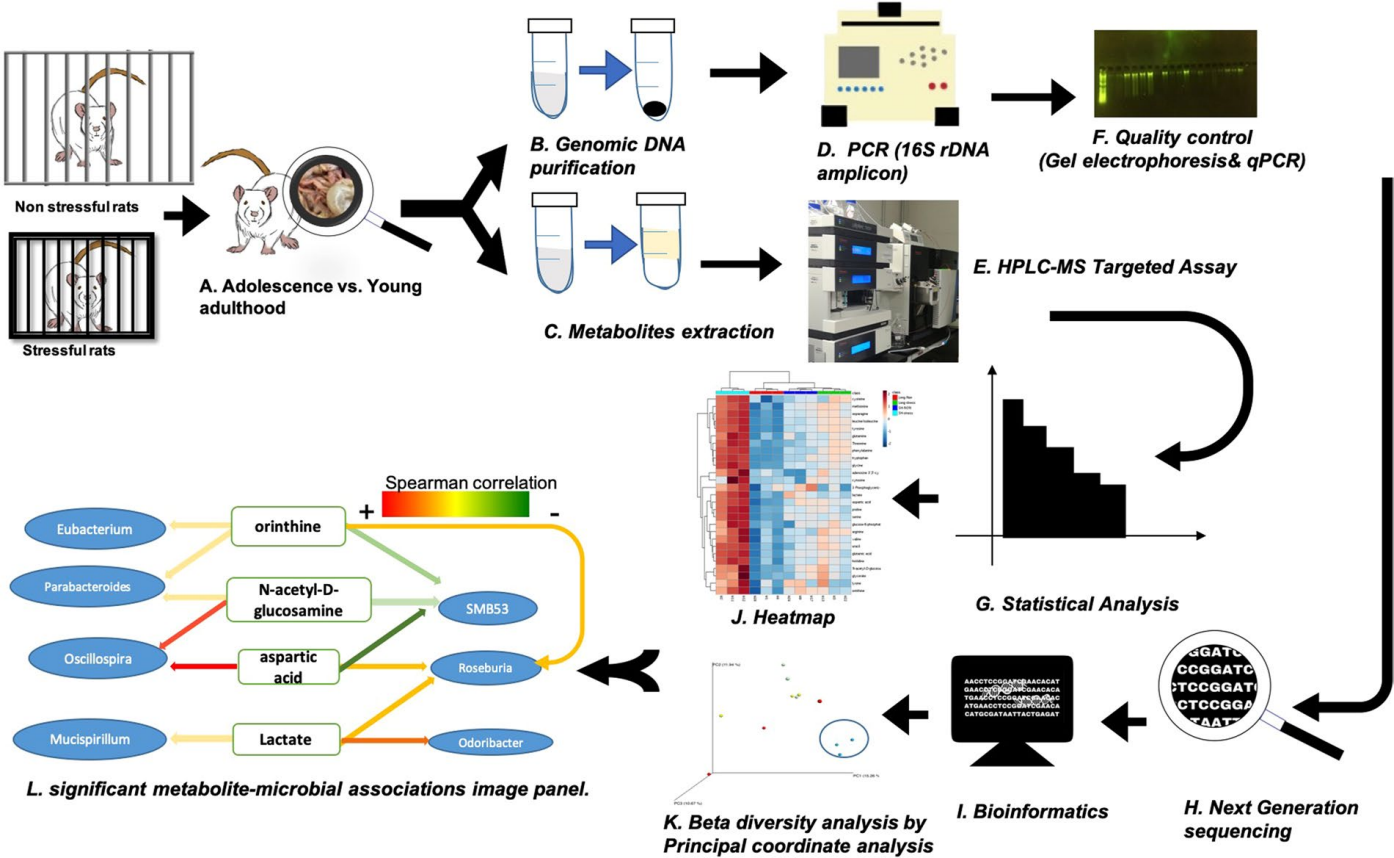
The workflow used in this study. (**A**) chronic restraint-induced stress was applied to Sprague Dawley rats during the critical adolescent period, while a control group remained in their home cages. Rats were sacrificed immediately after stress (PD 44) or 3 weeks after stress (PD 64) as a model of adolescent and young adulthood. (**B**) DNA isolation. (**C**) Gut microbial metabolites extraction. (**D**) PCR amplification of the 16S rDNA region (**E**) Apply targeted metabolic profiling for cecum samples. (**F**) The next generation sequencing library was quantitated by KAPA qPCR reaction and Agarose Gel Analysis were applied as quality control for size selection of genomic DNA. (**G**) Apply statistical analysis using MetaboAnalyst 4.0. (**H**) Next generation sequencing using the MiSeq platform. (**I**) Post-processing sequencing data were analyzed using QIIME. (**J**) Statistical analysis of metabolite profiles. (**K**) Principal co-ordinate plot represented the beta diversity of four groups. (**L**) Significant metabolite-microbial associations generated by Spearman correlation analysis.

Chronic restraint stress was used as a model to understand how stress during the critical adolescent period contributes to changes in the gut microbial population and metabolism. Twelve rats were used in this study. Following acclimation, rats were body-weight matched into stress and nonstress groups. Rats of the stress group were placed in cylindrical, Plexiglas restrainers (Plas-Labs, Lansing, MI, USA) for one-hour daily at random times during the light phase from PD 32 to PD 44 in adolescence^26^. The non-stress group remained in their home cages during times of stress. Body weight (BW) and food intake (FI) were measured throughout the experiment. Circulating levels of corticosterone (CORT), an indicator of HPA axis activation, were measured by ELISA (Enzo Life Sciences, Ann Arbor, MI, USA) using blood samples collected from the lateral tail vein of the 6 stress rats right before stress (baseline), 15 minutes, 30 minutes and 60 minutes during the one-hour restraint stress, as well as from the 6 nonstress rats at the same timepoints, on day 12 of chronic stress at PD 43. Half of the rats belonged to the adolenscent group that were sacrificed immediately after 13 days of the daily stress (S-Str) or control nonstress condition (S-Non) at PD 44; and the other half belonged to the adult group that were sacrificed 3 weeks after the stress (L-Str) or control condition (L-Non) at PD 64 when rats were young adults^26^, to determine if any gut microbial difference due to adolescent stress persisted into early adulthood. Sacrifices were performed by overdose of isofluranse anesthesia, and rat cecum samples immediately collected, weighed, and stored at −80 °C until further analyses. The workflow of the entire study is shown in Fig. 1.

### 16S rDNA sequencing

Microbial population analysis of the rat gut microbiome was done by 16S ribosomal DNA sequencing following a modified version of an established protocol published previously^16,27^. Cecal samples were isolated from rat cecum, and corresponding genomic DNA of these cecal samples was then isolated and purified with a commercialized kit (MP bio fast DNA spin kit for feces, Santa Ana, CA, USA). Purified genomic DNA samples were sent to Miami University Center for Bioinformatics and Functional Genomics for 16S rDNA sequencing. Breifly, the 515 f/806r primer set, pioneered by the Earth Microbiome Project (EMP; http://www.earthmicrobiome.org/), was used for PCR amplification of the 16S rDNA V4 region. Agarose gel electrophoresis was conducted for quality check of PCR products. Amplified 16S rDNA library was quantitated by Quan-iT Picogreen Assay (Quant-iT PicoGreen dsDNA Assay Kit, Thermo Fisher Scientific, Waltham, MA, USA) and quantitative PCR reaction (KAPA qPCR Illumina Universal Kit, Roche, Indianapolis, IN, USA). An Illumina Next Generation Sequencing MiSeq platform was used for amplicon sequencing. Raw Sequencing data was processed, cleaned, and analyzed using QIIME (1.9.1) software^28^. The barcode sequences of primers were used to assign sequencing reads back to each sample. In addition, quality-filtering of reads was done with default parameters of QIIME to improve diversity estimates^28,29^. An open-reference method was used to pick Operational Taxonomy Units (OTUs) against a 97% greengene database, a default of QIIME. An OTU table generated with the aforementioned method was used for downstream diversity analysis.

### Cecal sample metabolites extraction

Rat cecal metabolites were extracted based on a solvent-based method^30^. Briefly, the cecal sample isolated from the cecum were pre-weighted in a 2 mL Eppendorf tube. 1 mL of ice-cold methanol/chloroform (2:1, v/v) was added to each cecal sample. After homogenization on ice, samples were centrifuged for 10 minutes and the supernatant was collected for phase separation. Two phases of the sample were carefully collected separately into another set of 2 mL Eppendorf tubes. 0.5 mL of the polar phase was then concentrated by solvent evaporation in a concentrator. A mix of 50% acetonitrile and 50% water was used to reconstitute metabolites in LC vials and samples were then ready for metabolic profiling. All chemicals used were HPLC grade or DNA grade.

### Targeted HPLC-MS/MS metabolic profiling

The targeted metabolic profiling method used in this study was similar to our previous work^25,31^. Briefly, A Thermo Scientific Ultimate 3000 HPLC system was used to couple with TSQ Quantiva triple quadrupole mass spectrometer equipped with electrospray ionization source. A hydrophilic interaction chromatography column (Waters Corporation, Milford, MA, USA) was used for metabolite separation. The instrument was controlled by Xcaliber 2.0 software. Targeted data acquisition was performed in selected-reaction-monitoring (SRM) mode. The method was frequently validated using the same chemical standards during method set-up to ensure quality of the collected data. In case of ambiguity, the metabolite identities were confirmed by comparing peaks of the biological sample with mixtures of standard compounds for the expected signal increase at corresponding SRM transitions.

### Statistical analysis

BW % change, FI and CORT levels were analyzed using repeated measures two-way analysis of variance (ANOVA) with Bonferroni multiple comparison test that compared between nonstress and stress groups, and the areas under the curve for CORT measurements were analyzed using two-tailed unpaired t-tests assuming equal variance using Prism Statistical Software V8 (Graphpad Software, San Diego, CA). Quan Browser module of Xcalibur version 4.0 (Thermo Fisher Scientific) was used to process the raw metabolite data collected from HPLC–MS/MS analysis. Of the 55 targeted metabolites with selected amino acids, bases, nucleotides, organic acids, and metabolites related to frequently investigated pathways such as the TCA Cycle and glycolysis, 25 metabolites had detectable signals in more than 75% of the samples. Metabolite data were normalized using the weight of cecal components. MetaboAnalyst 4.0 (http://www.metaboanalyst.ca/) was used for statistical analysis following the recommended protocols^32^. A two-way ANOVA with false discovery rate correction was used to identify metabolite variabilities between different factors. Partial least squares discriminant analysis (PLS-DA) were applied for the comparison of metabolic profiles between four groups with *p-value* corrected for multiple testing (Bonferroni correction). Heat map presentation of intracellular metabolic profiles was also generated. The mass spectrum intensity values were log-transformed to approximate a normal distribution and auto-scaled. JMP Pro12 (SAS Institute, Cary, NC, USA) was used for Spearman correlation analysis between microbial genus abundances and metabolites in rat cecal samples. A built-in workflow of QIIME, core_diversity_analyses.py, was used for running a core set of QIIME diversity analysis with default parameters. P < 0.05 was considered to be statistically significant. Beta diversity was calculated as weighted and unweighted UniFrac distance in QIIME. To test the significant for the separation by beta diversity, both weighted and unweighted UniFrac distance were estimated by using a permutation analysis of variance (PERMANOVA) with the Adonis function in the “vegan” package in R.

## Results and Discussion

In this study, we aimed to evaluate the stress-induced microbial population and microbiota-related metabolic changes in an established rat model of chronic stress^33^. First, we confirmed the stress response using the circulating CORT levels during the stress test, as well as physiological parameters BW change and FI measures. As we expected, although the BW and daily FI were similar between nonstress and stress rats right before the chronic stress, BW % change of S-Str rats and FI of both S-Str and L-Str groups were significantly lower compared to their respective nonstress S-Non and L-Non rats on the last day of the treatment (p < 0.05, Table 1). BW % change had a trend to be different between L-Non and L-Str groups but did not reach statistic significance. On day 12 of the 13-day chronic stress, stress had significant overall effects on CORT levels, and CORT levels of stress rats were signigicantly higher than nonstress rats at 15 min timepoint (p < 0.05, Table 2). Additionally, the area under curve of CORT levels was significantly greater in stress rats than nonstress rats (p < 0.05, Table 2). Thus, the stress rats had elevated CORT levels and suppression in BW gain and feeding during the chronic stress. However, differences in BW change and feeding between stress and control groups were no longer present when the observation was extended for an additional three weeks after stress was ended and into early adulthood (Table 1).

**Table 1:** % Body weight change and food intake of stress and nonstress rats at different ages of postnatal day (PD).

Groups		S-Non	S-Str	L-Non	L-Str
Body weight change (%)	PD 31 (The day before stress)	0	0	0	0
	PD 44 (Last day of stress)	120.90 ± 5.7	77.60 ± 13.1*	107.69 ± 12.3	88.92 ± 7.8
	PD 64 (3 weeks after stress)	N/A	N/A	268.1± 15.5	252.7 ± 11.3
Feeding (g)	PD 31 (The day before stress)	21.69 ± 1.61	24.19 ± 2.27	21.09 ± 0.84	19.8 ± 0.36
	PD 44 (Last day of stress)	29.53 ± 0.07	19.78 ± 1.88*	26.13 ± 3.06	19.53 ± 0.75*
	PD 44–64 (3 weeks after stress)	N/A	N/A	599.56 ± 36.33	564.12 ± 15.30

*Indicates statistically significant difference between nonstress and stress groups (n = 3, p < 0.05).

**Table 2:** Plasma corticosterone (CORT) levels and area under curve of rats during one-hour control condition or restraint stress on day 12.

		Nonstress	Stress
CORT (ng/ml)	0 min (baseline CORT)	22.08 ± 6.00	19.95 ± 7.72
	15 min	53.03 ± 10.32	145.34 ± 14.54*
	30 min	73.68 ± 16.40	164.86 ± 29.77
	60 min	29.11 ± 10.32	97.53 ± 40.66
Area under curve		1008.47 ± 262.88	4276.29 ± 926.78*

*Indicates statistically significant difference between nonstress and stress groups (n = 6, p < 0.05).

**Figure 2:**
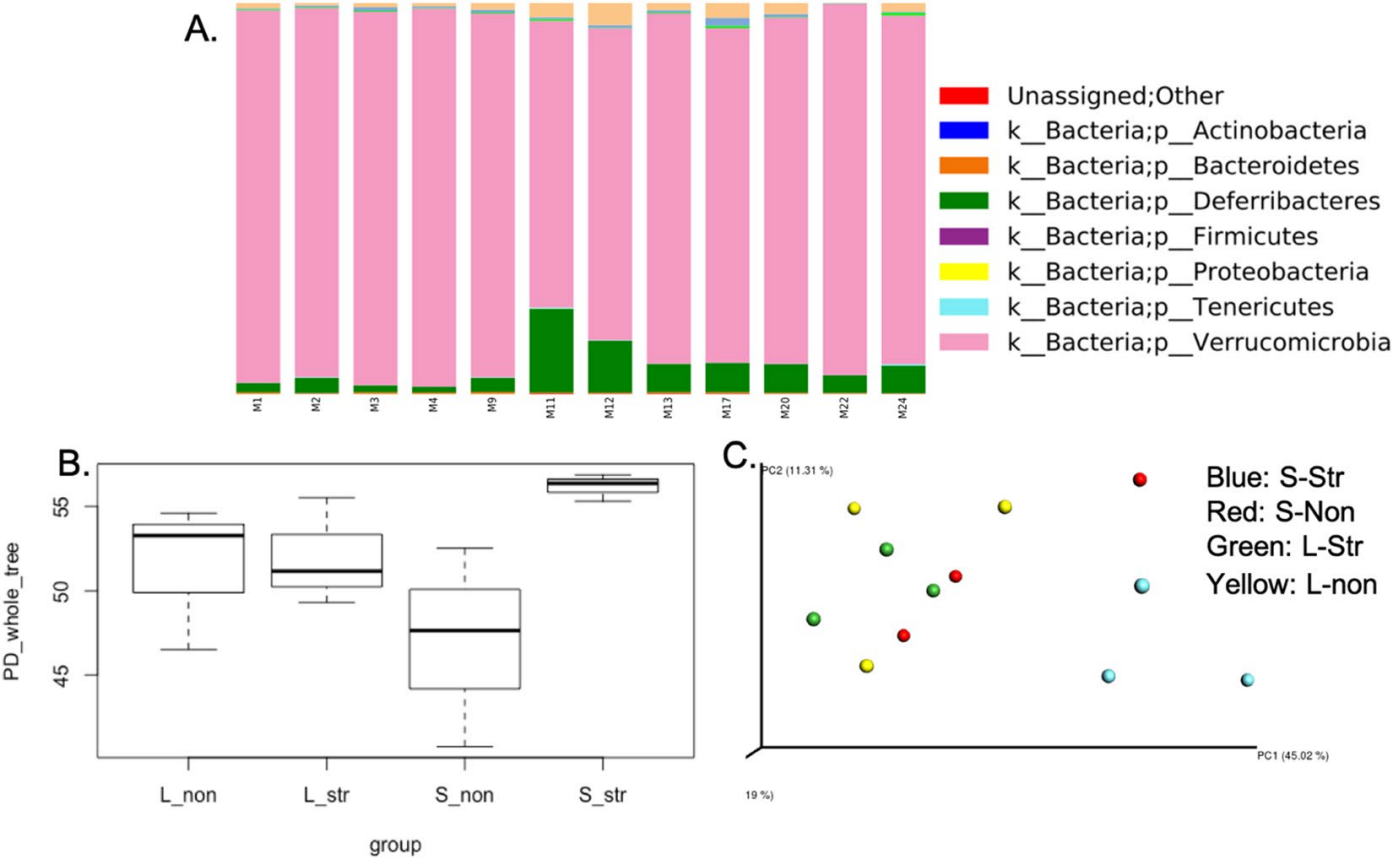
(**A**) Taxonomic composition of rat gut microbiota at the phylum level determined by 16S rRNA gene sequencing analysis. (**B**) The chao1 estimates. (**C**) Phylogenetic diversity (PD) estimates. (**D**) Total observed operational taxonomic units (OTUs). Alpha diversity analysis for rat gut microbiota from adulthood stressed group (L-Str), adulthood non-stressed group (L-Non), adolescent stressed group (S-Str), and adolescent non-stressed group (S-Non) were compared. € Beta diversity analysis by Principal coordinate analysis using an unweighted UniFrac metric.

Previous studies have revealed that the brain affects the community structure and function of the gut microbiota through the autonomic nervous system, leading to modulation of regional gut motility, intestinal transit and secretion, and gut permeability; and through the secretion of neurohormones that directly modulate microbial gene expression^34–36^. Therefore, it is reasonable to expect that chronic daily restraint stress of this study would impact microbial genes and metabolism, at least during the treatment period. Therefore, we examined microbes collected from the cecum samples of all 12 rats belonging to four different experimental groups with different ages (adolescent vs. adult) and treatments (stress vs. no stress) to evaluate the stress-induced gut microbial population shift and their metabolic differences. Adolescent group were sacrificed immediately after 13 days of the daily stress (S-Str) or control nonstress condition (S-Non) at PD 44; Adult group were sacrificed 3 weeks after the stress (L-Str) or control condition (L-Non) at PD 64 when rats were young adults^26^. The taxonomic overview of the gut microbial composition at the phylum level revealed 7 include *Actinobacteria, Bacteroidetes, Deferribacteres, Firmicutes, Proteobacteria, Tenericutes*, and *Verrucomicrobia* were found which was consistent with the constitution of mammal gut microbiota from a published report^37^. Moreover, 11 classes, 15 orders, 26 families, and 32 genera were identified. However, there was no strong characteristic composition pattern among different treatment groups. Further analysis evaluated alpha diversity of different groups with Faith’s phylogenetic diversity (Fig. 2B). The indice indicated differences between S-Str vs. S-Non, but not between L-Str vs. L-Non. Faith’s phylogenetic diversity analysis indicated that S-Str displayed significantly elevated species richness (56.18 ± 0.80) compared to S-Non (46.97 ± 5.92), L-Non (51.46 ± 4.33), and L-Str (52.00 ± 3.19) (Fig. 2C). To further elucidate differences among treatment groups, structures of these microbes were assessed by beta diversity analysis (unifrac) (Fig. 2C). The resulting principal coordinate analysis plot clearly presented that microbiota of S-Str rats were well clustered and separated from the other three groups. A PERMANOVA test was performed and resulted in significant differences for separation from S-Str to other group by using weighted UniFrac distance measurement (Table S1).

**Figure 3:**
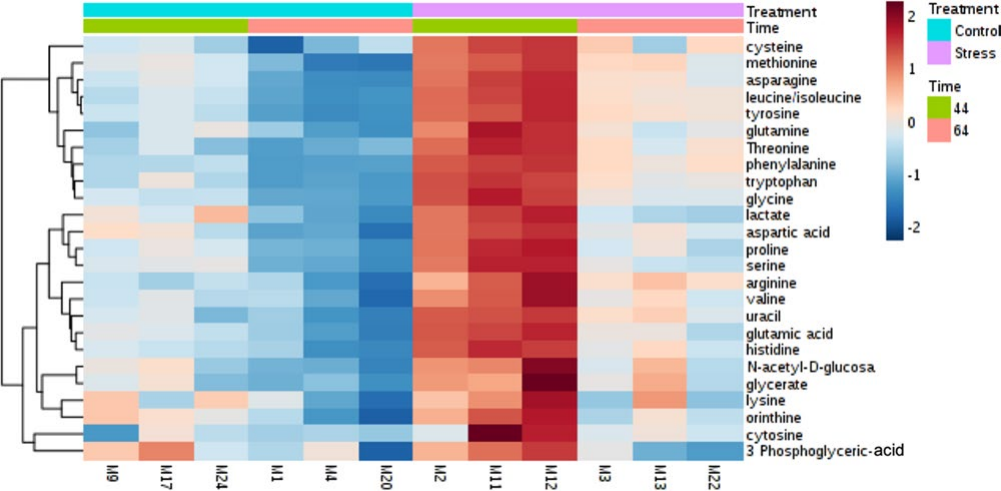
Heat map presentation of intracellular metabolic profiles from adult non-stressed group (L-Non), adult stressed group (L-Str), adolescent non-stressed group (S-Non), and adolescent stressed group (S-Str). Each column represents one biological replicate, and each row represents one targeted metabolite detected in this study.

**Figure 4:**
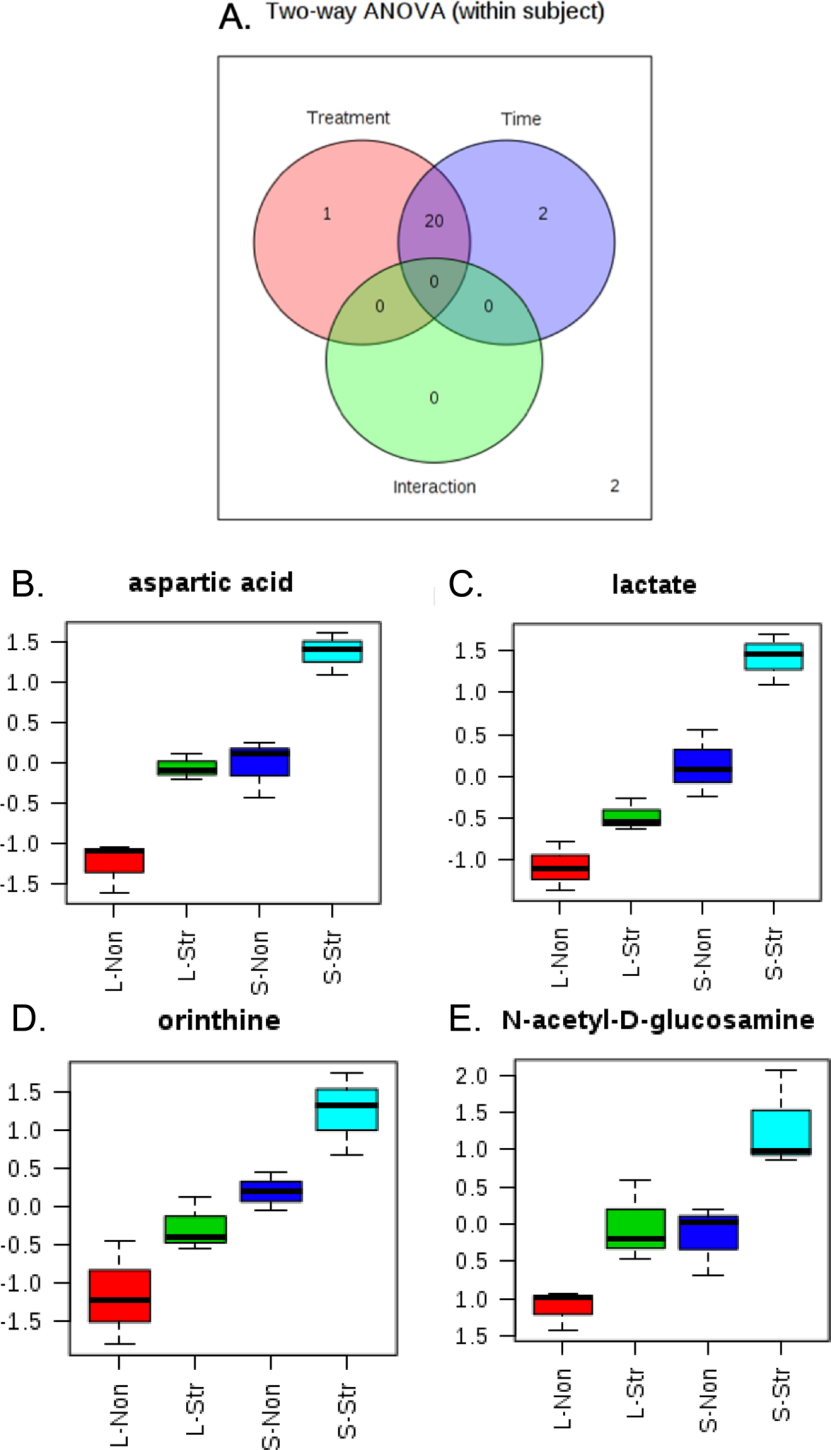
(**A**) Number of significant metabolites detected by repeated measure two-way ANOVA with False discovery rate correction. 23 metabolites that have a significant difference (*p* < 0.005) among the four groups comparison with two factors in this study. 21 The following panels presented four selected examples of these metabolites. (**B**) aspartic acid; (**C**) lactate; (**D**) ornithine; (**E**) N-acetyl-D-glucosamine.

We then performed metabolomics analysis to further investigate the metabolic changes of these gut microbes under different stress conditions. Metabolites were extracted from the cecum samples based on the established liquid extraction method^38^. A total of 25 metabolites were detected in the majority of the samples using our targeted metabolomics approach and were used for statistical analyses. In the heat map showing the profile of detected metabolites (Fig. 3), each column represented one biological replicate and each row represented an individual metabolite detected. The figure indicated clustering of the four cohorts and demonstrated the ability of metabolic for separation. Two-way ANOVA discovered 23 out of 25 metabolites being significantly different among four groups (*p* < 0.005; Fig. 4A). 21 metabolites were significantly affected by Stress and 22 metabolites were significantly affected by age. 20 metabolites were identified as affected by both stress and age. Four representative metabolites, aspartic acid (Fig. 4B), lactate (Fig. 4C), ornithine (Fig. 4D), and N-acetyl-D-glucosamine (Fig. 4E) that have 3 or more correlation to bacterial genus (see below discussion for details) were graphed as examples to show the differences among four groups where S-Str has the highest production and L-Non has the lowest production on all of the four metabolites.

**Figure 5:**
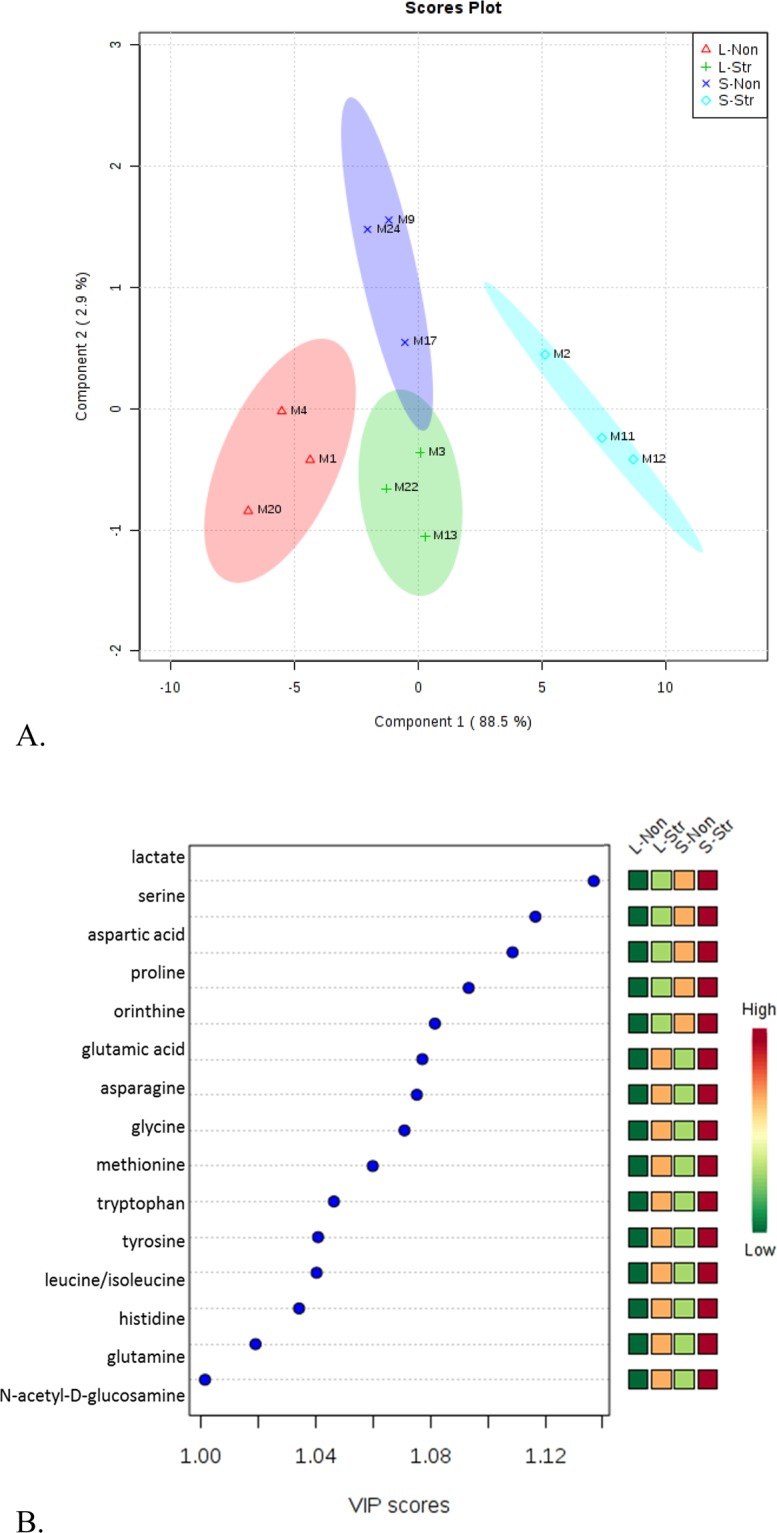
(**A**) Partial Least Squares Discriminant Analysis (PLS-DA) of 25 metabolites data differentiates adolescent stressed group (S-Str), adult non-stressed group (L-Non), adolescent non-stressed group (S-Non) and adult stressed group (L-Str) based on their metabolic profiles. (**B**) Variance importance projection (VIP) plot shows the top 15 metabolites that contribute to the separation of groups by PLS-DA approach. The contribution can be quantified by VIP scores that listed at the X-axis.

An overview of the entire metabolic profile from each sample was generated using partial least square-discriminant analysis (PLS-DA) to confirm whether the metabolic activities of of the four groups (L-Non, L-Str, S-Non, and S-Str) were different. The metabolic profiles of S-Non and S-Str groups were clearly separated (Fig. 5A). These results supported our hypothesis that stress affects the gut microbial population and consequently their metabolic profiles in rats during adolescence; however, the effect on gut microbial population did not extend after stress into early adulthood. The list of the 15 metabolites that contributed to the group separation in Fig. 5A was arranged from top-to-bottom (Fig. 5B) based on the rank of their variance importance projection (VIP) scores. The top fifteen metabolites were all detected in greater abundance in S-Str group compared to other groups. These included those amino acids and non-amino acid metabolites involved in major pathways, such as the TCA cycle and glycolysis. While these metabolites were increased by the presence of chronic stress during the adolescent period, decreases of these metabolites were observed after chronic stress was ended, three additional weeks into early adulthood.

Stress, a ubiquitous part of human daily life, is well-known to exert varied biological effects, including modulation of gut microorganisms residing in the gastrointestinal tract and consequent changes in their relative populations and metabolic functions^21,39^. In turn, the gut microbiota can influence the host stress response through at least three parallel and interacting channels involving nervous, endocrine, and immune signaling mechanisms, thereby implicating the gut microbiota as an important mediator of host health^17,40^. It is unclear if such influence is temporary or persistant. There are more than four decades of literature showing the effect of stress on the gut microbial community structure^17^; however, the effect of stress on microbial metabolic process at different post-stress timepoints has rarely been studied. This pilot study examined the chronic stress-induced gut microbial population and metabolic responses immediately following adolescent stress and examined if any of these changes persisted long-term into early adulthood. The findings from our study indicated that clear differentiation of both microbial population and metabolism can be observed immediately after 13 days of one hour-daily restraint stress treatment. It is interesting that the significant difference in microbial population diminished three weeks after the stress was removed (Fig. 2), while the microbial metabolic profiles remain distinguishable between the group that had gone through L-Str and L-Non at this time point (Figs. 3 and 4). These results suggest that the possible recovery of stress-induced dysbiosis is a faster process than the recovery of stress-induced gut microbial metabolic functionality. Although this study analyzed a limited number of animals and further investigation is needed to validate the conclusion, this promising finding of time-dependent recovery of gut microbial functions changed by stress-related events will provide preliminary rationales for many future investigations.

**Figure 6:**
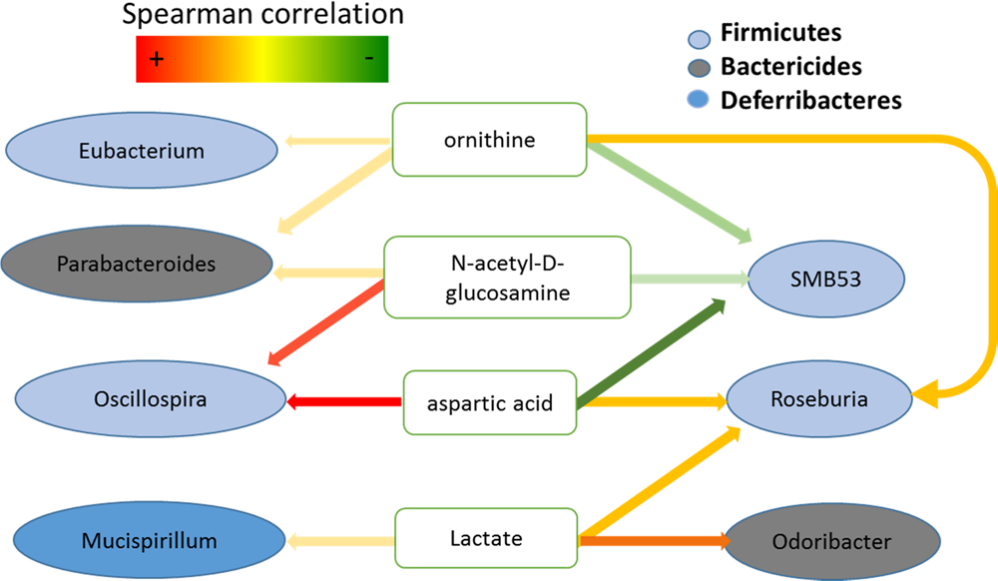
(**A**) diagram indicates the relationships among the top 4 metabolites (white rounded rectangles) that have 3 or more correlation to different bacterial genus (colored ovals).

In addition to the separate analyses of microbial genomics and microbial metabolites, we also intended to understand their connections through correlation analysis. The metabolites peak intensity and relative microbial genus abundances were used to compute the spearman rank-based correlation matrix. By applying a spearman correlation cutoff of 0.65, 8 bacteria genera and 20 metabolites were found to be associated with one another. The most frequently correlated 4 metabolites that have 3 or more correlation with bacterial genus and their correlated 7 genera (colored spheres in Fig. 6)reflected a total of 13 significant types of microbe-metabolites correlation. The different colors of the bacterial genus represented the phylum, where each significantly correlated with the source of genus (Fig. 6). Two dominant bacterial phyla identified in this study were *Firmicutes* and *Bacteroidetes* (Fig. 2). It is interesting to note that all bacterial genus in phyla *Bacteroidetes* displayed positive associations with the four representative metabolites listed in Fig. 4. However, the bacteria genera in phyla *Firmicutes* demonstrated both positive and negative associations with correlated metabolites. A strong association between the amount of aspartic acid and the abundance of *Oscillospira* was found. Positive associations between N-acetyl-D-glucosamine, ornithine, and lactate and *Eubacterium, Parabacteroides, Mucispirillum, Roseburia* and *Odoribacter* were revealed in this study. In addition, negative associations between aspartic acid, N-acetyl-D-glucosamine, and ornithine with SMB53 were also observed in this study. Similarly, rats with chronic stress exposure had increased levels of aspartic acid, N-acetyl-D-glucosamine, ornithine, and lactate; the former three being negatively associated with bacteria genus SMB53. In addition, higher levels of aspartic acid and N-acetyl-D-glucosamine, that have a strong positive correlation with bacteria genus *Oscillospira*, were detected immediately after the chronic stress exposure.

Based on a thorough literature review, some of our results and observations are completely new findings, while several other findings can be supported by previous reports in the literature. For example, previous studies have revealed that *Eubacterium* in phyla *Firmicutes* is generally considered a health-promoting gut microbe^41,42^, and this bacterial genus was detected at relatively decreased abundance in the S-Str compared to other groups, which echos the fact that stress generates adverse health effects to the gut microbes. It is also interesting to note that the lactate level of rats’ cecum samples significantly elevated immediately after the stress event in this study, which could contribute to gut dysbiosis and increased host gut permeability and inflammation^43^.

There is an increasing recognition that humans are ‘super-microorganisms’ comprised of an integrated network of gut microbes, whose dynamic bidirectional interactions with human host play an important role in stress response and host health. It is pivotal for us to understand the microbial population and metabolic adaptation of the host during stress events, and to generate intervention recommendations in the future to promote the gut and host health. Our pilot study utilized rodent restraint stress and 16S rDNA sequencing to provide preliminary, first-hand evidence of different post-stress recovery rates between the gut microbiome population and those same populations’ altered metabolism due to stress. This paves the way for future studies of the microbiome in order to promote stress-related gut and host health. One caveat of our study is that litter cannot be accounted for in the analysis of microbiome, given the way the rats were acquired. We also fully acknowledge that our study has several limitations, including relatively small sample size and limited coverage in metabolite detection, and we are confident that we will be able to address these challenges in our future studies.

## Supplementary information

Supplementary information.
