# Supplementary material for: Difference in post-stress recovery of the gut microbiome and its altered metabolism after chronic adolescent stress in rats
Source: Sci Rep. 2020 Mar 3;10:3950. doi: 10.1038/s41598-020-60862-1 (PMC7054252; doi:10.1038/s41598-020-60862-1)
Supplement: Supplementary file 1 — Supplementary information. [file 41598_2020_60862_MOESM1_ESM.docx]

**Supplementary information for:**

**Difference in post-stress recovery of the gut microbiome and its altered metabolism after chronic adolescent stress in rats**

Mengyang Xu^1^, Chen Wang^1^, Kristen Krolick^1^, Haifei Shi^1^ and Jiangjiang Zhu ^2, 3^*

1. Department of Biology, Miami University, Oxford, OH 45056, USA

2. Department of Human Sciences, The Ohio State University, Columbus, OH 43210, USA

3. James Comprehensive Cancer Center, The Ohio State University, Columbus, OH 43210 USA

*Corresponding author.

Dr. Jiangjiang Zhu, Department of Human Sciences and James Comprehensive Cancer Center, The Ohio State University, Columbus, OH 43210, USA

Email: [zhu.2484@osu.edu](mailto:zhu.2484@osu.edu)

Table S1. Model results for both weighted and unweighted UniFrac distance metrics are provided.


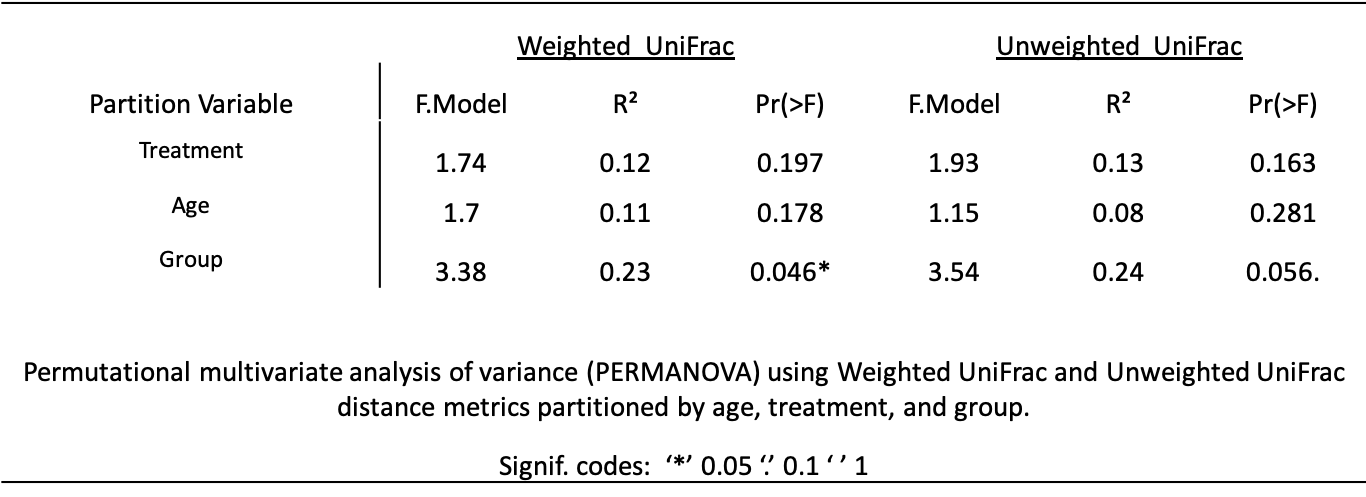


Table S2. 23 metabolites that significantly affected by either treatment of time were found by repeated measurement two-way ANOVA(p<0.05).

| Metabolites | Treatment(adj.p) | Time(adj.p) | Interaction(adj.p) |
| --- | --- | --- | --- |
| leucine/isoleucine | 8.02E-05 | 0.0024648 | 0.40617 |
| phenylalanine | 8.02E-05 | 0.0018119 | 0.1315 |
| glycine | 8.02E-05 | 0.0018119 | 0.1315 |
| tyrosine | 8.02E-05 | 0.0024648 | 0.49028 |
| asparagine | 0.000108 | 0.0032541 | 0.49028 |
| serine | 0.0001756 | 0.0032541 | 0.3543 |
| glutamine | 0.0001756 | 0.021797 | 0.42174 |
| Threonine | 0.00037071 | 0.011355 | 0.27401 |
| tryptophan | 0.00050589 | 0.0024648 | 0.27401 |
| methionine | 0.00076677 | 0.0056761 | 0.91029 |
| lactate | 0.0017598 | 0.004522 | 0.42174 |
| proline | 0.0022091 | 0.0032541 | 0.27401 |
| glutamic acid | 0.0028644 | 0.0032541 | 0.27401 |
| histidine | 0.0038267 | 0.0024648 | 0.27401 |
| uracil | 0.0043493 | 0.0056761 | 0.40617 |
| aspartic acid | 0.0052361 | 0.0018119 | 0.49028 |
| cysteine | 0.005592 | 0.043259 | 0.49028 |
| arginine | 0.0057898 | 0.032821 | 0.67953 |
| valine | 0.0072375 | 0.019404 | 0.49028 |
| glycerate | 0.0088628 | 0.07743 | 0.67953 |
| N-acetyl-D-glucosamine | 0.0094573 | 0.029224 | 0.67953 |
| orinthine | 0.055321 | 0.0039874 | 0.76434 |
| 3 Phosphoglyceric-Acid | 0.48767 | 0.013235 | 0.49028 |
